# Phyto-Carbazole Alkaloids from the Rutaceae Family as Potential Protective Agents against Neurodegenerative Diseases

**DOI:** 10.3390/antiox11030493

**Published:** 2022-03-01

**Authors:** Mario A. Tan, Niti Sharma, Seong Soo A. An

**Affiliations:** 1 College of Science and Research Center for the Natural and Applied Sciences, University of Santo Tomas, Manila 1015, Philippines; matan@ust.edu.ph; 2 Department of Bionano Technology, Gachon University, 1342 Seongnam-daero, Sujeong-Gu, Seongnam 461-701, Korea

**Keywords:** neurodegenerative disease, Rutaceae, phyto carbazole alkaloid, oxidative stress, neuroinflammation, Alzheimer’s disease

## Abstract

Plant-derived (phyto) carbazole alkaloids are an important class of compounds, presented in the family of Rutaceae (Genera *Murraya*, *Clausena*, *Glycosmis*, *Micromelum* and *Zanthoxylum*). Due to several significant biological activities, such as antitumor, antibacterial, antiviral, antidiabetic, anti-HIV and neuroprotective activities of the parent skeleton (3-methylcarbazole), carbazole alkaloids are recognized as an important class of potential therapeutic agents. Neurodegenerative diseases (NDs) may exhibit a vast range of conditions, affecting neurons primarily and leading ultimately to the progressive losses of normal motor and cognitive functions. The main pathophysiological indicators of NDs comprise increasing atypical protein folding, oxidative stresses, mitochondrial dysfunctions, deranged neurotransmissions and neuronal losses. Phyto-carbazole alkaloids can be investigated for exerting multitarget approaches to ameliorating NDs. This review presents a comprehensive evaluation of the available scientific literature on the neuroprotective mechanisms of phyto-carbazole alkaloids from the Rutaceae family in ameliorating NDs.

## 1. Introduction

Neurodegenerative diseases (NDs) belong to a heterogeneous groups of disorders (Alzheimer’s, Parkinson’s, multiple sclerosis, amyotrophic lateral sclerosis, Huntington’s disease, etc.), which could result in progressive loss of structure or function of the neurons. These neurological changes in the brain could lead to logical and/or functional deterioration over time. The main physiological indicators of NDs comprise increasing atypical protein misfolding, oxidative stress, mitochondrial dysfunction, deranged neurotransmission and neuronal loss [1,2].

Alzheimer’s disease (AD) is the most prevalent form of ND and is described by declining cognitive and motor functions. Currently, the available drugs for the treatment of AD are acetyl cholinesterase (AChE) inhibitors (donepezil, galantamine and rivastigmine) or blockers of glutamate receptors (memantine). Recently, the Food and Drug Administration (FDA) approved an antibody drug (Aduhelm), which may improve AD symptoms. Yet, all these allopathy drugs have associated side effects, ranging from minor headache to swelling and brain haemorrhaging [3]. In this regard, plants could provide an effective and safer source of bioactive compounds to be used as a drug. Researchers have identified several plants with neuroprotective properties, like AChE inhibition, retarding atypical aggregation and scavenging free radicles, which protect against oxidative stress [3,4,5]. Additionally, several bioactive compounds have anti-inflammatory properties and reduce the activity of pro-inflammatory markers (interleukins (IL-6, IL-1β), nitric oxide (NO), tumor necrosis factor-α (TNF-α) and inflammatory proteins inducible nitric oxide synthase (iNOS) and cyclooxygenase-2 (COX-2)) [3,6].

Carbazole alkaloids are characterized by a tricyclic aromatic basic skeleton consisting of a central pyrrole ring fused with two benzene rings. Phyto-carbazoles are basically derived from 3-methyl carbazole as the common precursor. Carbazoles are privileged scaffolds, often reported in natural products [7], and exhibit a wide spectrum of biological activities, such as anticancer [8], neuroprotective [9], antituberculosis [10], and anti-HIV (Human Immunodeficiency Virus) [11]. Two drugs, Midostaurin and Carvediol, with a carbazole core were approved by the FDA for treating tumors and congestive heart failure, respectively [12]. 

Phyto-carbazole alkaloids are abundantly found in species of the Rutaceae family, including *Murraya koenigii* (Curry leaves), *Glycosmis pentaphylla* (Gin berry), *Clausena heptaphylla* (Clausena), *Clausena excavate* (Pink Lime-Berry)*, Murraya euchrestifolia*, *Micromelum* sp. and *Zanthoxylum* sp. [13]. Among the naturally occurring phyto-carbazole alkaloids, mahanimbine, koenimbine, koenigicine and clausazoline-K were reported to possess anti-lipase activity [14,15], while mahanine, pyrayafoline-D and murrafoline-I displayed anticancer activities by inducing apoptosis through activating the caspase-9/caspase-3 pathway [16]. Both natural and synthetic derivatives of carbazole alkaloids have revealed numerous pharmacological activities, such as anticancer [17], antioxidant [18], anti-inflammatory [19], antibacterial [20], antifungal [21], antidiabetic [22], antiangiogenic [23], larvicidal [24], anti-plant virus [25], anti-HIV [26], and neuroprotective activities [13]. In particular, carbazole-containing arylcarboxamides and carbazole thiazoles are inhibitors of β-secretase (BACE-1: the enzyme responsible for the production of β-amyloid (Aβ)) and Aβ formation, respectively [27,28]. Several dibenzofuran/carbazole derivatives inhibit acetylcholinesterase (AChE) and Aβ aggregations [29]. Owing to strong antioxidant and neuroprotective effects towards neurons displayed by N-substituted carbazoles [30], hybrids comprising tacrine and carbazole were developed, with anti-AChE activities [9]. 

**Figure 1:**
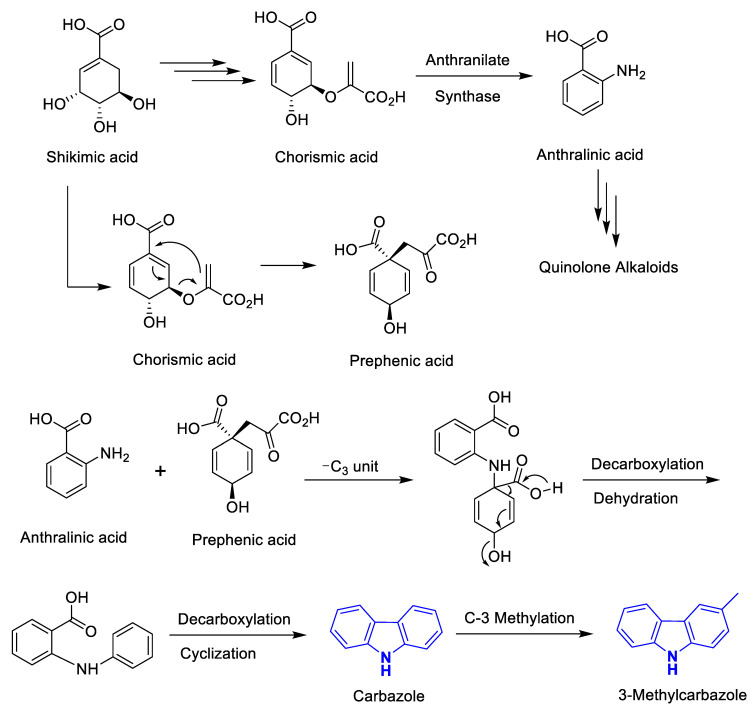
Biogenetic pathway in the formation of carbazole and 3-methyl carbazole alkaloids and core structure of the diverse carbazole alkaloids isolated from nature.

Biogenetically, the phyto-carbazole alkaloids are postulated to originate from the shikimate pathway (Figure 1) [31,32]. As shown in Figure 1, the diverse phyto-carbazole alkaloids identified from nature are classified based on their carbazole or 3-methylcarbazole core structure. Next, in vivo oxidation of the methyl group of the 3-methylcarbazole provides the formyl carbazole or methyl carbazole-3-carboxylate congener structures, which are commonly found in the genera *Murraya*, *Clausena* and *Glycomis* [31]. Moreover, the majority of the more than 330 identified phyto-carbazole alkaloid derivatives from nature are based on 3-methylcarbazole as their common precursor [13].

Due to the immense medicinal properties of phyto-carbazole alkaloids, the present review aims to highlight the pharmacological action mechanisms of important and prevalent phyto-carbazoles from the Rutaceae family in treating the pathophysiology of NDs. Owing to the complex etiology of NDs, a multitarget approach of bioactive compounds in ameliorating disease symptoms may achieve better clinical results. Hence, this review could be a basis for potential future complementary treatment of complex NDs.

## 2. Phyto-Carbazole Alkaloids in Neuroprotection

### 2.1. Phyto-Carbazole Alkaloids from the Genus Murraya

*Murraya koenigii* is a rich source of phyto-carbazole alkaloids, isolated from the leaves, stem, bark, and root, with promising pharmacological activities [33,34]. Neuroprotective pharmacological activities of the *M. koenigii* leaf (MKL) extract are also associated with phyto-carbazole alkaloids. When total alkaloidal extract of MKL was administered orally to different groups of mice, a significant improvement in memory scores was observed in the elevated plus maze and passive avoidance apparatus models [35]. Interestingly, reductions in the brain cholinesterase activity (~20% reduction) and BACE-I activity (IC_50_ 1.7 µg/mL) were also observed with MKL alkaloids [35]. The actual phyto-carbazole alkaloids in the above study are yet to be identified, but euchrestine B, bismurrayafoline E and (+)-mahanine exhibited potent antioxidant activities in 2,2-diphenyl-1-picrylhydrazyl (DPPH) assay (21.7 μM, 6.8 μM and 21.9 μM, respectively) in comparison to the standards tocopherol, butylated hydroxytoluene (BHT) and ascorbic acid (27.8 μM, 83.2 μM and 4.4 μM, respectively) [36].

The anti-amnesic potential of MKL was observed in mice fed MKL powder mixed feed [37]. Murrayakonine A, *O*-methylmurrayamine A and mukolidine isolated from *M. koenigii* leaves exhibited strong anti-inflammatory effects against lipopolysaccharide (LPS) induced human peripheral blood mononuclear cells (PBMCs) by reducing the production of TNF-α and IL-6 [38]. Murrayanol also presented anti-inflammatory potential against human prostaglandin-endoperoxide H synthase (hPGHS-1) (IC_50_ 109 µg/mL) and hPGHS-2 (IC_50_ 218 µg/mL) [39]. 

Murrayamine-E (at 10 μM), isolated from *M. koenigii*, showed substantial effects on neurite outgrowth. For exploiting the phyto-carbazole skeleton of murrayamine-E as a potential pharmacophore, similar derivatives were synthesized. One of the compounds, 9-benzyl-9*H*-carbazol-4-ol, revealed robust influences on neurite outgrowth. In addition, it improved memory in APdE9 mice. These results suggest that carbazole derivative 9-benzyl-9*H*-carbazol-4-ol would be an important compound for ND drug discovery [40].

Microphyltrine, microphyldines A-O and microphyldine P were isolated from the stem and leaves of *M. microphylla* [41]. Nitric oxide production induced by LPS in BV-2 microglial cells was inhibited by phyto-carbazole alkaloids from *M. tetramera* and *M. kwangsiensis* [19,42], with IC_50_ values ranging from 5.1 to 15.1 μM [43]. 

Mahanimbine, a major phyto-carbazole alkaloid from *M. koenigii*, was reported as an in vitro inhibitor of AChE (IC_50_ 0.03 mg/mL) [44]. It also displayed high antioxidant activity (IC_50_ 33.1 µg/mL) in comparison to the positive controls: *tert*-butylhydroquinone (TBHQ), BHT and butylated hydroxyanisole (BHA) (IC_50_ 3.3, 4.4 and 18 μg/mL, respectively) [39]. 

In vivo and in vitro studies confirmed the beneficial approach of the multi-target properties of mahanimbine in AD treatment, which presented its neuroprotective potentials in SK-N-SH neuroblastoma cells against 100 μg/mL LPS. Interestingly, the pre-treatment of SK-N-SH cells with mahanimbine significantly prevented cell loss and attenuated LPS-induced reactive oxygen species (ROS) formation. Additionally, mahanimbine also inhibited BACE 1 with IC_50_ of 4 µg/mL. From an in vivo study, the biochemical analysis of the whole brain of ICR mice detected increased CAT and GRD levels and significant decreases of malondialdehyde (MDA) levels in mahanimbine-treated groups in comparison to the untreated group [45]. 

In LPS-challenged mice, mahanimbine improved cognition in the Morris water maze (MWM) experiment. It also improved the central cholinergic transmission by increasing acetylcholine (ACh) levels in the brain homogenate (27.48 ± 2.44 μM, 24.40 ± 2.87 μM, 23.19 ± 2.62 μM) through AChE inhibition. Additionally, mahanimbine greatly reduced Aβ_1-40,_ pro-inflammatory cytokines (IL-1 β and TNF-α), the total activity of COX, and expression of the COX-2 gene in LPS-induced group. These findings support the neuroprotective activity of mahanimbine against LPS-induced neuroinflammation [46]. It was observed that mahanimbine reduced oxidative stress through nuclear factor erythroid-2-related factor 2 (Nrf2)-dependent induction of antioxidant enzymes (SOD and CAT) and by subduing the expressions of proinflammatory cytokines (Nuclear factor kappa: NF-κB, TNF-α, IL-1β) and Aβ accumulations [35,47,48,49].

Murrayanine is the first reported phyto-carbazole alkaloid from the stem bark of *M*. *koenigii* with potential antioxidant properties [50]. When anti-inflammatory effects were investigated both in vitro and in vivo, a reduction of NO (a prominent pro-inflammatory molecule released in both acute and chronic inflammatory conditions) was observed [51]. In addition, pro-inflammatory cytokines (IL-6 and TNF-α) were also decreased in both LPS-stimulated RAW 264.7 cells and murine peritoneal macrophages. Moreover, iNOS and COX-2 protein expressions, as well as their downstream product, prostaglandin E2 (PGE2), were also decreased effectively in murine macrophage RAW 264.7 cells. Murrayanine also suppressed the inhibitor of nuclear factor kappa B (I-κβ) phosphorylation and NF-κB activity in LPS-activated RAW 264.7 cells. The NF-κβ pathway is important for preserving synaptic plasticity and balancing between learning and memory. Therefore, any impairment in the pathways associated with NF-κβ signaling cause altered neuronal dynamics [52]. Additionally, administration of murrayanine in a systemic inflammation mouse model inhibited pro-inflammatory cytokines and also increased the survival rate in LPS-challenged mice.

From in silico studies using random forest (RF) models, several phytocompounds were evaluated for their interaction potentials against COX-1 and COX-2 through molecular docking, dynamics simulation and free energy calculations [53]. Girinimbine from *M. koenigii* was selective towards COX-2, which was supported by the experimental studies of inhibiting COX in an anti-inflammation model. Cytotoxicity assessment of girinimbine against the breast cancer cell line MDA-MB-231 also supported it as a COX-2 inhibitor (IC_50_ 0.006 µg/mL) [53].

Furthermore, girinimbine inhibited inflammation in vitro as well as in vivo. In LPS/INF-γ induced RAW 264.7 cells, significant dose-dependent girinimbine inhibitions of NO production and NF-κβ translocation from the cytoplasm to nucleus were observed [54]. Girinimbine revealed its considerable antioxidant activity, equivalent to 82.17 ± 1.88 μM of Trolox at 20 μg/mL. In a carrageenan-induced peritonitis mouse model, oral pre-treatment of girinimbine reduced pro-inflammatory cytokine levels (IL-1β, TNF-α) in the peritoneal fluid [54]. In a mice model of ethanol-induced gastric ulcers, GSH and MDA levels were restored in the girinimbine treatment group with decreased levels of proinflammatory cytokines (TNF-α and IL-6) and iNOS. Girinimbine could also selectively inhibit COX-2 [55]. Still, no study has reported the direct neuroprotective effects of girinimbine. The anti-inflammatory and antioxidant potential of girinimbine in neurodegenerative diseases should be investigated. 

**Figure 2:**
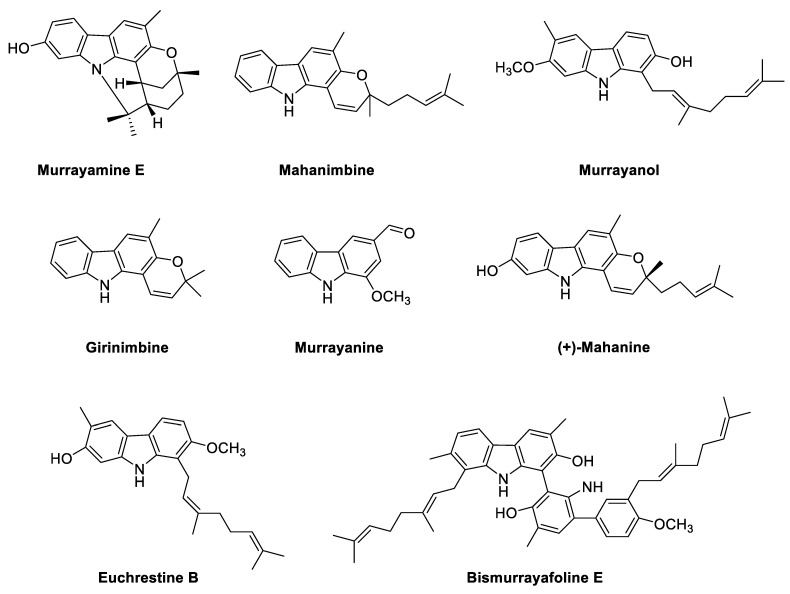
Potential neuroprotective phyto-carbazole alkaloids from *Murraya koenigii*.

Structures of the phyto-carbazole alkaloids isolated from *M. koenigii* with potential neuroprotective effects against neurodegenerative diseases are presented in Figure 2.

### 2.2. Phyto-Carbazole Alkaloids from the Genus Clausena

**Figure 3:**
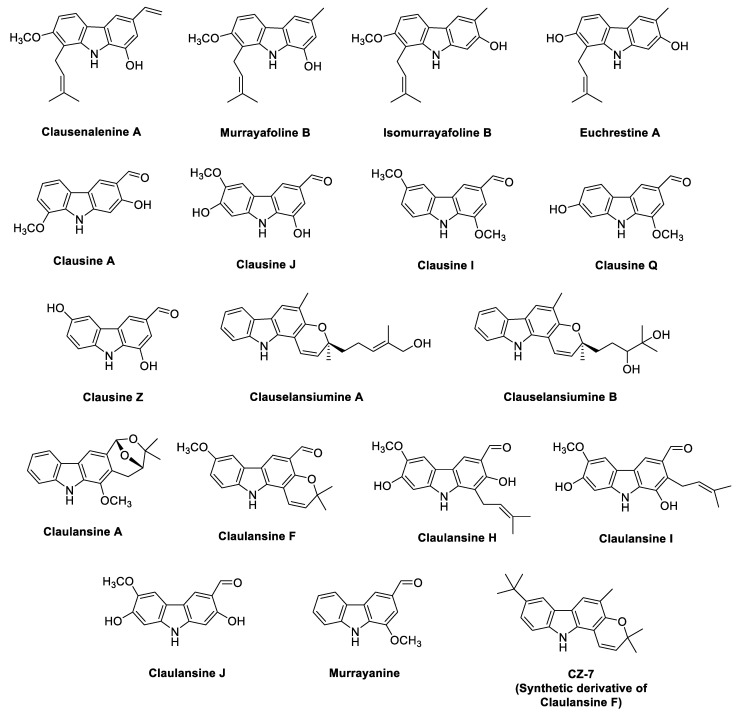
Potential neuroprotective phyto-carbazole alkaloids from the species of the genus *Clausena*.

Structures of the phyto-carbazole alkaloids isolated from various *Clausena* species with potential neuroprotective effects against NDs are presented in Figure 3. Phyto-carbazole alkaloid clausenanisines A–F and clausevestines were isolated from the fruits of *C. anisumolens* [56] and from the stem and leaves of *C. vestita* [57], respectively. Various prenylated carbazole alkaloids presented a significant inhibition (IC_50_ 0.63–8.3 μM) of NO production in mouse macrophage RAW 264.7 cells by LPS induction, with hydrocortisone (IC_50_ 3.8 μM) as the positive control [57,58]. Clauemarazoles A–G and its derivatives were isolated from the stems of *C. emarginata*. Among them, clauemarazole E, clausine K and clausine O exhibited inhibitory abilities on LPS-induced NO production (IC_50_ 10.91, 4.6 and 6.4 μM, respectively) [59]. Still, no direct study has been conducted on the neuroprotective potential. Similarly, phyto-carbazoles from *C. dunniana* showed weak inhibitory effects on NO production stimulated by LPS in BV-2 microglial cells (IC_50_ > 50 μM) [60].

Since many extracts from *C. lansium* possessed strong antioxidant activities in vitro [61], the neuroprotective and cognitive enhancing effects of various concentrations of peel crude extract were investigated on a rat model of focal cerebral ischemia. A significant reduction of brain infarction and oxidation indices, a restoration of memory, a revived level of antioxidants, and enriched survival and cholinergic neurons were witnessed in the hippocampal region in vivo [61].

Hydroethanolic extract of the stems and leaves of *C. lenis* exhibited significant neuroprotective activities against the induced apoptosis with 6-hydroxydopamine (6-OHDA) in human neuroblastoma SH-SY5Y cells (SH-SY5Y, EC_50_ 12.86 μg/mL). Eight phyto-carbazole alkaloids—clausenalenine A, murrayafoline B, isomurrayafoline B, euchrestine A, clausine A, clausine J, clausine I and clausine Q—possessed substantial neuroprotective effects (EC_50_ 0.68 to 18.76 μM) against 6-OHDA-induced apoptosis in SH-SY5Y by 6-hydroxydopamine. Hence, regular consumption of these fruits may protect against Parkinson’s disease (PD) [62].

Clausine Z from *C. excavata* showed potent inhibition of recombinant Cyclin-dependent kinase 5 (CDK5) (IC_50_ 0.51 mM) in a filter plate assay in comparison to the standard inhibitor: butyrolactone I (IC_50_ 0.11 mM). In cell-based studies, clausine Z protected cerebellar granule neurons against free radical-induced apoptosis, with an EC_50_ of 1.1 mM in comparison to the standard butyrolactone I (EC_50_ 3 mM) [63]. Studies have revealed that neuroprotection is correlated with the inhibition of neuron-specific CDK5 [64]. For neurite outgrowth and cortical lamination, CDK5 and its neuron-specific activator p35 are essential [65]. Proteolytic cleavage of CDK 5 activator p35 by calpain led to the formation and accumulation of p25 in the brain of AD patients [66]. This proteolytic cleavage resulted in prolonged activation of cdk5 and subsequently hyper-phosphorylation of tau by the p25/cdk5 kinase, in turn disrupting the cytoskeleton and promoting apoptosis of primary neurons [67]. The p25 is neurotoxic in nature and results in apoptosis [68]. Moreover, it is seen as a downstream regulator of Aβ [67]. Thus, small molecule inhibitors of CDK5 could be important contenders for the therapeutic development of neurodegenerative diseases, as they protect against p25 neurotoxicity [69]. Hence, clausine Z and derivatives may present important therapeutic values in neurodegenerative diseases, such as AD, PD and amyotrophic lateral sclerosis (ALS).

Geranylated phyto-carbazole alkaloids (clauselansiumines A–B) along with other carbazole alkaloids from the stem and leaves of *C. lansium* revealed remarkable neuroprotective effects (EC_50_ 0.48 to 12.36 μM) against 6-OHDA-induced apoptosis in SH-SY5Y cells [70]. The protective effect of phyto-carbazoles (10 μM) was also reported on primary neurons against oxygen glucose deprivation (OGD) injury [71].

Ten new phyto-carbazole alkaloids (claulansines A–J) and their analogues were isolated from *C. lansium* and were assessed for their neuroprotective effect [72]. Claulansine A, F, H–J, and murrayanine were found to be neuroprotective at 10 μM [72]. All these alkaloids displayed prominent neuroprotective activity (EC_50_ 0.36 to 10.69 μM) in comparison to the standard curcumin (EC_50_ 5.8 μM) in 6-OHDA-induced apoptosis in SH-SY5Y cells [73]. Structure activity relationship (SAR) demonstrated the importance of the aldehyde group or hydroxymethyl group at C-3 position in noteworthy neuroprotective effects. Among them, claulansine F (Clau F), a pyrano[3,2-a] carbazole alkaloid, revealed protection against sodium nitroprusside (SNP)-induced apoptosis in pheochromocytoma (PC12) cells [74], which exerted a significant effect on hydroxyl free radical scavenging and mitochondrial integrity. Based on the encouraging neuroprotective activity of claulansine F, its analogues were synthesized, and their neuroprotective effects were studied against hydrogen peroxide (H_2_O_2^−^_) and OGD-induced injury in PC12 cells and primary cortical neurons [75]. The SAR showed a stronger neuroprotective effect when the methyl group was present at C-3 and at C-6, with no substitution at N-9, in comparison to the *N*-alkyl substitution. In contrast, with the presence of aldehyde at C-3 and the same group at C-6, *N*-alkyl substitution exhibited much stronger neuroprotective effects than without substitution at N-9. In addition, lipophilic groups at C-6 displayed stronger activities (CZ-7, with a *t*-Bu at C-6). CZ-7 exhibited the strongest neuroprotective effects in vitro and at lower doses efficiently protected against ischemic stroke in a rat model of Middle Cerebral Artery Occlusion (MCAO). Furthermore, CZ-7 showed stronger free radical scavenging capacity than Edaravone (EDA), which was therapeutically used as a strong antioxidant and neuroprotective agent in ALS. Most importantly, CZ-7 could pass through the blood-brain barrier (BBB) in rats with 4.3-fold higher concentrations in brain than plasma [75]. These results suggest the importance of groups at C-3, C-6 and N-9; the lipophilic groups in the compounds are the crucial factors for neuroprotective activity of carbazoles [75].

In another study, oral treatment of CZ-7 ameliorated cognitive impairment in rats with permanent occlusion of bilateral common carotid arteries (2VO) [73]. Morris water maze tests revealed that CZ-7 considerably reduced the escape latency in 2VO rats. Morphological studies using Nissl and terminal deoxynucleotidyl transferase dUTP nick end labelling (TUNEL) staining showed that the administration of CZ-7 markedly diminished the pathological changes in the CA1–CA3 area of the hippocampus, including neuronal cell loss, nuclear shrinkage, and dark staining of neurons, and significantly decreased chronic cerebral hypoperfusion-induced cell loss. Additionally, CZ-7 significantly improved the white matter lesions as seen in Klüver-Barrera staining. CZ-7 administration significantly decreased oxidative stress in the CA1–CA3 area of the hippocampus from 8-hydroxydeoxyguanosine (8-OHdG) and ROS immunofluorescent analyses. The double immunofluorescent staining of Nrf2 and the elevated expressions of oxidative stress proteins Heme oxygenase-1 (HO-1) and NAD(P)H: quinone oxidoreductase 1 (NQO1) suggested that CZ-7 recovered the oxidative stress through the Nrf2 pathway [73].

Clau F-donepezil (a FDA-approved AChE inhibitor) hybrids were synthesized through additional development to evaluate the neuroprotective potential. The benzylpiperidine fragment of donepezil interacted with the catalytic domain of AChE. For free-radical scavenging activity, the indanone moiety was replaced by Clau F (or its analogue CZ-7 fragments). The Clau F–donepezil hybrids exhibited potent AChE inhibition (IC_50_ 1.63–4.62 μM) [73]. Furthermore, (*E*)-3-(8-(tert-Butyl)-3,3-dimethyl-3,11-dihydropyrano[3,2-a] carbazol-5-yl)-N-((1-(2-chlorobenzyl) piperidin-4-yl) methyl) acrylamide (Compound 6bd) demonstrated superior neuroprotective effects in comparison to Clau F against OGD/reoxygenation (OGD/R). Additionally, the compound 6bd displayed good BBB penetration (Permeability: Pe × 10^−6^ cm·s^−1^) in parallel artificial membrane permeation assay (PAMPA) [25].

### 2.3. Phyto-Carbazole Alkaloids from Glycomis pentaphylla

**Figure 4:**
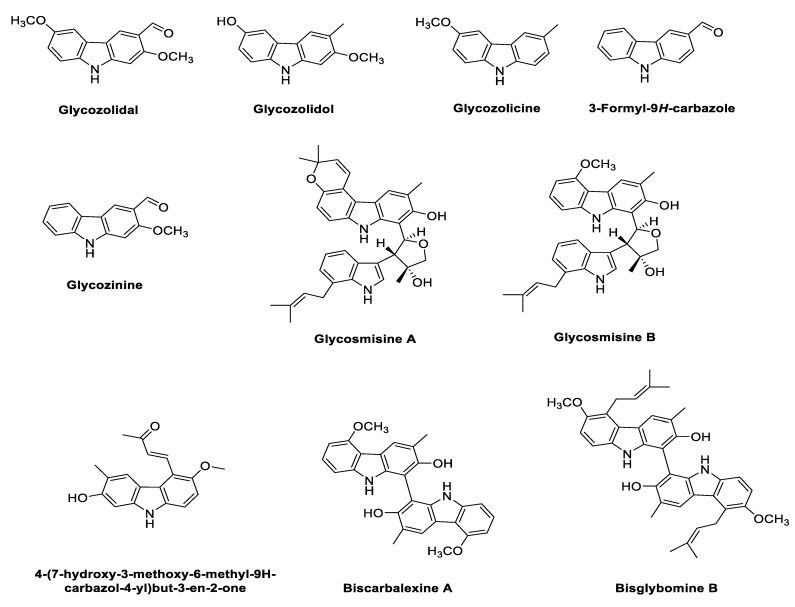
Potential phyto-carbazole alkaloids from *Glycomis pentaphylla*.

The genus *Glycosmis* is considered as a rich source of potentially biologically active secondary metabolites, such as alkaloids, flavonoids, phenolic glycosides, quinones and terpenoids. Plants in the genus *Glycosmis* were used in traditional medicine for the treatment of various diseases, like anxiety, cancer, snake bites and joint pain [76,77,78,79]. Structures of the phyto-carbazole alkaloids isolated from *G. pentaphylla* are presented in Figure 4.

Phytochemical analyses shows the isolation and elucidation of glycozolidol, glycozolicine, 3-formyl-9*H*-carbazole and glycosinine from the roots of *G. pentaphylla* [80,81,82], while 4-(7-hydroxy-3-methoxy-6-methyl-9H-carbazol-4-yl)but-3-en-2-one, bisglybomine B, biscarbalexine A, carbazole–indole-type dimeric alkaloids, and glycosmisines A and B were isolated from the stem of *G. pentaphylla* [83,84].

The methanolic extract of *G. pentaphylla* exhibited significant dual AChE (IC_50_ 325.1 ± 0.91 μg/mL) and butyryl cholinesterase (BChE) effects (IC_50_ 42.1 ± 3.30 μg/mL). Furthermore, the extract showed radical scavenging ability in 2,2-diphenyl-1-picrylhydrazyl (DPPH) assay (IC_50_ 95.6 ± 0.68μg/mL) and a lipid peroxidation inhibitory effect (IC_50_ 288.7 ± 0.91 μg/mL) [85]. The total crude alkaloid extract of *G. pentaphylla* showed antioxidant potential in the DPPH assay (IC_50_ 966.93 µg/mL), ferric reducing antioxidant power (FRAP) (IC_50_ 510.81 µg/mL), 2,2′-azino-bis (3-ethylbenzothiazoline-6-sulfonic acid) (ABTS) assay (IC_50_ 400.47 µg/mL), hydroxyl free radical (OH·) assay (IC_50_ 1805.28 µg/mL) and NO assay (IC_50_ 1426.50 µg/mL). Thus, these phyto-carbazole alkaloids were the potential therapeutic target for NDs such as AD, PD and other oxidative stress-related diseases [86].

### 2.4. Phyto-Carbazole Alkaloids from Micromelum

**Figure 5:**
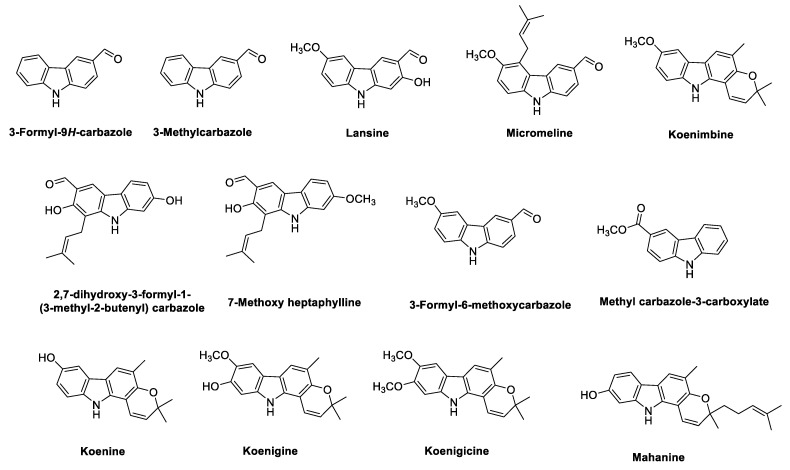
Potential phyto-carbazole alkaloids from the species of the genus *Micromelum*.

*Micromelum* is a rich source of bioactive secondary metabolites. Methylene chloride extract of the stem bark of *M. hirsutum* is rich in phyto-carbazole alkaloids, including 3-methylcarbazole, 3-formylcarbazole, lansine, micromeline, 3-formyl-6-methoxycarbazole and methyl carbazole-3-carboxylate, and some of these have displayed anti-tuberculosis activity [87]. Structures of the phyto-carbazole alkaloids isolated from the species of the genus *Micromelum* are presented in Figure 5.

Two phyto-carbazole alkaloids, 2,7-dihydroxy-3-formyl-1-(3-methyl-2-butenyl) carbazole and 7-methoxy heptaphylline, have been isolated from the roots of *M. glanduliferum*, which are used as chemotaxonomic markers to differentiate this plant from other *Micromelum* species [88]. Koenimbine, koenine, koenigine and koenidine were isolated from the leaves and young stems of *M. zeylanicum* [89]. Additionally, mahanine from the leaves of *M. minutum* presents vast therapeutic effects, such as anticancer, anti-mutagenicity, antimicrobial and anti-inflammatory [90,91]. Phyto-carbazole alkaloids glycozolinol and methyl carbazole-3-carboxylate were isolated from the leaves of *M. integerrimum* [92]. However, in spite of the immense pharmaceutical importance of phyto-carbazole alkaloids from *Micromelum*, no study has been conducted on the neuroprotective action of *Micromelum* species [93].

### 2.5. Carbazole Alkaloids from Zanthoxylum

**Figure 6:**
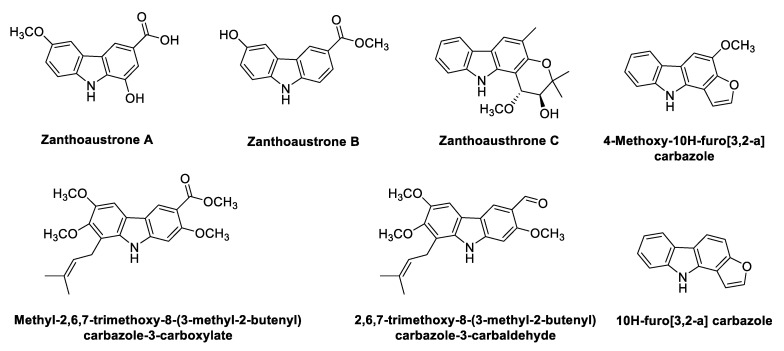
Potential neuroprotective phyto-carbazole alkaloids from the species of the genus *Zanthoxylum*.

*Zanthoxylum* species (syn. *Fagara* species) are widely used as food and in traditional systems of medicine for treating inflammation, pain, hypertension and neurological diseases [94]. Structures of the phyto-carbazole alkaloids isolated from *Zanthoxylum* species with potential neuroprotective effects against NDs are presented in Figure 6. The phytochemicals responsible for the biological activities of some of the species have yet to be identified.

Isolated Zanthoaustrones A–C from the roots of *Z. austrosinense* [95] significantly inhibited NO production (IC_50_ 1.59 ± 0.11, 1.29 ± 0.06, 0.89 ± 0.05 μM, respectively) in comparison to hydrocortisone (IC_50_ 4 μM). Two prenylated alkaloids 2,6,7-trimethoxy-8-(3-methyl-2-butenyl) carbazole-3-carbaldehyde and methyl-2,6,7-trimethoxy-8-(3-methyl-2-butenyl) carbazole-3-carboxylate were isolated from ethyl acetate soluble fraction of *Z. armatum*, which demonstrated substantial antioxidant potential in DPPH free radical scavenging assay [96]. From the ethanol-soluble extract of *Z. fagara* bark, two novel furocarbazole alkaloids with antibacterial activity, 4-methoxy-10H-furo[3,2-a] carbazole and 10H-furo[3,2-a] carbazole, were isolated [96,97]. Han et al. [98] evaluated the antioxidant, antidiabetic, and neuroprotective activity against high glucose–induced cytotoxicity of *Z. piperitum* (ZP) and *Z. schinifolium* (ZS) extracts. The extracts displayed strong antioxidant potential in ABTS/DPPH assays, and MDA contents were significantly reduced. ZP inhibited carbohydrate hydrolysis (α-glucosidase and α-amylase) more efficiently than ZS in antidiabetic tests. Interestingly, ZS in comparison to ZP decreased anti-advanced glycation end-products (AGE) more effectively. AGEs have an important role in the progression and pathogenesis of AD, as Aβ aggregation is accelerated in the presence of AGEs [99]. The content of AGEs is more in neurofibrillary tangles (NFTs) and plaques, as suggested by immunohistochemical studies [100]. Additionally, both ZP and ZS effectively protect human-derived neuronal cells from high glucose-induced cytotoxicity, indicating the neuroprotective nature of the plants. In a recent study [101], pericarp of *Z. schinifolium* (ZSP) unveiled effective DPPH (IC_50_ 75.6 ± 6.1 µg/mL) and ABTS (IC_50_ = 57.4 ± 6.0 µg/mL) radical scavenging activities. ZSP also inhibited the release of pro-inflammatory cytokines, IL-1β (IC_50_ 134.4 ± 7.8 µg/mL), IL-6 (IC_50_ 262.8 ± 11.2 µg/mL) and TNF-α (IC_50_ 223.8 ± 5.8 µg/mL).

The methanolic and ethyl acetate extracts of *Z. capense* root exhibited neuroprotective effects in rotenone-elicited neuronal injury in SH-SY5Y. Pre-treatment of SH-SY5Y cells with the extracts significantly reduced ROS generation and improved intracellular glutathione levels. Moreover, the extracts inhibited rotenone-induced activation of caspase-3 and subsequent apoptosis. Comparatively, methanol extract displayed better neuroprotective activity than ethyl acetate extract [102].

*Z. bungeanum* is another popular spice in East and Southeast Asia [103], which is used to treat forgetfulness and other symptoms in Chinese traditional medicine [104]. Different *Z. bungeanum* extracts (water, volatile oil, petroleum ether and methylene chloride) were prepared to evaluate its role in cognitive improvement in D-galactose-induced aging mice [105]. The weakened memory was considerably alleviated after water and volatile oil extract treatment. These extracts also protected against neuron damage in the hippocampus by D-galactose induction. Additionally, treatment with water and volatile oil extracts of *Z. bungeanum* facilitated the recovery of oxidative stress parameters (SOD, CAT, GSH, MDA) and oxidative stress response genes (*Nrf2* and *HO-1*) in the mouse brain. Furthermore, activation of the phosphoinositide 3-kinase (PI3K)/protein kinase B (Akt) pathway increased the expression of B-cell lymphoma 2 (Bcl2)-associated X apoptosis regulator (Bax), with a concomitant reduction in the expression of (Bcl2) in the mouse brain [105]. The important role of the PI3K/AKT signalling pathway in neurogenesis, neuronal proliferation and synaptic plasticity is very well ellucidated [106]; it also regulates the expression of Bcl-2, Bax and other related proteins [107]. Additionally, the activation of the PI3K/AKT pathway promotes the growth of dopamine neurons by inhibiting apoptosis [108,109], thereby playing a neuroprotective role in the treatment of AD and PD.

Activation of Nrf2 is an attractive target for the prevention of AD [110], as its levels are reported to decrease in AD. Hence, it could be suggested that *Z. bungeanum* extracts are promising agents for the prevention of aging-related cognitive dysfunction and neurological deficits [105]. Additionally, the ethyl acetate fraction of the *Z. bungeanum* leaf exhibited the strongest ABTS and DPPH radical scavenging activities in comparison to chloroform, water fractions and crude extract in vitro. The ethyl acetate fraction protected PC12 cells against hydrogen peroxide–induced cytotoxicity [111]. Similarly, methanol:chloroform (1:4) extract of the *Z. piperitum* leaf was a potent radical scavenger and reducing agent, which also displayed protective effects against H_2_O_2_-induced neurotoxicity in a concentration-dependent manner in PC12 cells [112]. 

Different species of *Zanthoxylum* (*Z. fagara, Z. rhoifolium, Z. monophylla and Z. quinduensis)* exhibited considerable antioxidant and AChE inhibitory potential (116.8, 107.4, 10.2 and 123.0 µg/mL, respectively) in comparison to the standard, galantamine (4 µg/mL) [113], which was due to the presence of alkaloids and other phytochemicals. In addition, methanolic and ethyl acetate root extracts of *Z. davyi* displayed potent anti-AChE activity (IC_50_ 0.01 ± 0.004 mg/mL and 0.011 ± 0.002 mg/mL, respectively) [114].

Another study elaborated the role of hydro ethanolic extract of *Z. alatum* (HEZA) in ameliorating scopolamine-induced amnesia in rats by targeting multiple pathways for cognition enhancement [115]. HEZA displayed AChE inhibition, antioxidant effects and inhibition of neuroinflammation (TNF-α, IL-1 β and IL-10) in the hippocampus. Pre-treatment with HEZA considerably down-regulated the expression of *NF*κ*B*, *Tau*, Bax and Caspase-3, with simultaneous up-regulation of *Nrf2*, *HO-1*, *PP2A* (serine/threonine protein phosphatase 2A), BDNF (brain-derived neurotrophic factor) and TrkB (Tropomyosin receptor kinase B) genes in the hippocampal region [115]. HEZA exhibited antioxidant activity through up-regulating *Nrf2*-mediated *HO*-*1* expressions. The phosphorylation and dephosphorylation of *Tau* were regulated by many proteinases, such as *PP2A*. It was demonstrated that endogenous *PP2A* was reversibly inhibited during oxidative stress [116]. Next, since BDNF is an important manager of synaptogenesis and synaptic plasticity, the increased levels of BDNF by HEZA could help with memory enhancement [117]. Hence, *Z. alatum* extract revealed positive effects on cognition, and it is a potential candidate for drug development for NDs.

Interestingly, the majority of the identified phyto-carbazole alkaloids in Figure 2, Figure 3, Figure 4, Figure 5 and Figure 6 originated from 3-methylcarbazole as their mutual precursor, and the oxidation product of 3-methylcarbazole resulted in 3-formyl or 3-carboxyl structures (Figure 2, Figure 3, Figure 4, Figure 5 and Figure 6) [13,31,32]. The predominant number of the derived phyto-carbazoles from 3-methylcarbazole [13] include the C-_13_-type 3-methylcarbazoles, represented by 3-methylcarbazole, glycozolidol and glycozolicine; the C-_13_-type 3-formylcarbazoles, such as 3-formylcarbazole, lansine, glycozine and murrayanine; the C-_13_-type 3-carboxylcarbazole derivatives, including methyl carbazole-3-carboxylate and zanthoaustrone A and B; the C-_18_-type 3-methylcarbazole alkaloids, such as girinimbine, koenine, koenigine, euchrestine A and murrayafoline B; the C-_18_-type 3-formylcarbazoles, represented by 7-methoxyheptaphylline, claulansine F, and claulansine H; the C-_18_-type 3-carboxylcarbazoles, represented by methyl-2,6,7-trimethoxy-8-(3-methyl-2-butenyl)carbazole-3-carboxylate; the C-_23_-type 3-methylcarbazoles, such as mahanine and mahanimbine; and the C-_23_-type 3-formyl derivatives. Dimeric phyto-carbazole alkaloids, including bismurrayafoline E, glycosmisine A and B, biscarbalexine A and bisglybomine B, were also reported as potential neuroprotective agents from the genus Rutaceae. With the diversity of the phyto-carbazole structures reported (Figure 2, Figure 3, Figure 4, Figure 5 and Figure 6), the identification of a specific pharmacophore responsible for the reported biological activities, including neuroprotective effects, may be a challenge. However, the presence of the aldehyde or hydroxymethyl group at the C-3 position may contribute to the neuroprotective activity, based on a previous structure activity relationship (SAR) study [74]. An SAR report on the synthetic derivatives of claulansine F (Figure 3) showed a stronger neuroprotective effect with methyl groups at C-3 and C-6 positions. *N*-9-alkylation resulted in decreased activity. In comparison, substitution of an aldehyde group at C-3 and C-6 positions, including a *N*-9-alkylated moiety, showed stronger neuroprotective effects. Thus, groups at C-3, C-6 and N-9, along with the lipophilic groups, were crucial for neuroprotective activity of the carbazole [75] as specified in Section 2.2. However, a deeper understanding of SAR studies of the isolated phyto-carbazole alkaloids from nature would be worth exploring. 

## 3. Conclusions and Future Perspectives

**Figure 7:**
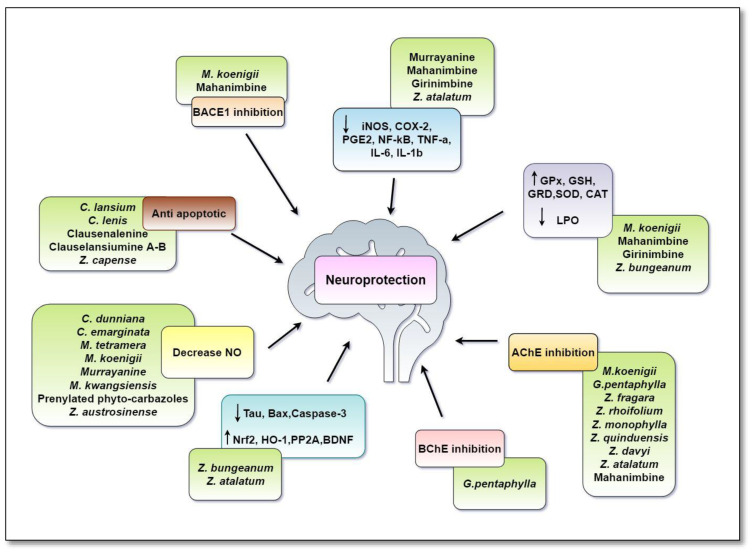
Multi-target mechanism displayed by Rutaceae family plant extracts and carbazole alkaloids in neurodegenerative diseases. Phyto extracts and carbazole alkaloids from Rutaceae family exerted a multi-target approach in ameliorating symptoms of AD. *PP2A* and BDNF activity/expression was decreased in *AD*, which stimulated the hyper-phosphorylation of Tau and APP, leading to overproduction of Aβ. Phyto-carbazoles prevented Aβ aggregation by inhibiting cleavage of the APP by BACE-I, as well as by upregulating the expression of PP2A and BDNF. This caused a shift in the non-amyloidogenic pathway and reduced the levels of produced Aβ. A number of phyto-carbazoles were shown to reduce the levels of pro-inflammatory cytokines and inflammatory mediators (IL-6, IL-1β, NF-kβ, TNF-α, iNOS, COX-2). Nrf2/HO-1 was an antioxidant signaling pathway, which was required for neuronal cell proliferation and survival. Down-regulation of this pathway was associated with various NDs. Phyto-carbazoles provided the neuroprotection by upregulating the expressions of the Nrf2/OH-1 pathway. They also reduced the oxidative stress by increasing the levels of antioxidant enzymes (SOD, CAT, GSH, GRD, GPx) and reducing lipid peroxidation (LPO). Caspase-3 and Bax were responsible for neuronal death and were up-regulated in AD. Phyto-carbazoles down-regulated their expressions and prevented apoptosis. ACh, a neurotransmitter essential for processing memory and learning, was decreased in both concentration and function in AD. Decreased levels of ACh could be restored by the AChE and BChE inhibitory activity of phyto-carbazole alkaloids. Overproduction of NO was linked to neuroinflammation, mitochondrial dysfunction and neurotoxicity in NDs. The increased levels of NO were also controlled by phyto-carbazole and plant extracts from the Rutaceae family. Abbreviations: Aβ: Amyloid beta; ACh: Acetylcholine; APP: Amyloid Precursor Protein; AChE: Acetyl Cholinesterase Enzyme; BACE 1: Beta-Secretase 1; Bax: BCL2 Associated X; BChE: Butyl Cholinesterase Enzyme; BDNF: Brain-Derived Neurotrophic Factor; CAT: Catalase; COX-2: Cycloxygenase-2; GSH: Reduced Glutathione; GRD: Glutathione Reductase; GPX: Glutathione Peroxidase; HO-1: Heme Oxygenase-1; iNOS: inducible Nitric Oxide Synthase; IL: Interleukin; NO: Nitric Oxide; NF-kβ: Nuclear Factor Kappa B; Nrf2: Nuclear factor erythroid 2–Related Factor 2; PP2A: Protein Phosphatase 2A; SOD: Superoxide Dismutase; TNF-α: Tumor Necrosis factor-α.

Researchers all over the world are aiming to benefit from the immense natural chemical diversity of plants against a diversity of pharmacological targets. A number of accomplishments have been documented in finding new entities from the nature; several candidates have been further developed into new drugs, while others have served as a stepping stone for further drug discovery. Table 1 and Figure 7 summarize the neuroprotective activities of Rutaceae family plant extracts.

The present review discussed the neuroprotective potential of various phyto-carbazole alkaloids, exclusively present in the Rutaceae family plants, such as *Murraya*, *Clausena*, *Glycosmis*, *Micromelum* and *Zanthoxylum*. The discovery of the first naturally occurring phyto-carbazole alkaloid, murrayanine, from *M. koenigii*, is considered as a landmark in the progress of the chemistry and biology of carbazoles [31]. Phyto-carbazole alkaloids are important moieties in drug discovery and display a range of therapeutic activities, like anticancer, anti-tuberculosis, anti-HIV, antibacterial and neuroprotective.

In the past few decades, the therapeutic importance of natural and semi-synthetic carbazole alkaloids has expanded considerably. The phyto-carbazole alkaloids manifest the neuroprotection using a multi-target approach, lowering the oxidative stress, attenuating proinflammatory cytokines, inhibiting AChE and BACE-1, preventing/reducing Aβ protein aggregation and improving cognition. These pathophysiological features are common to almost all NDs. Unfortunately, the currently available FDA-approved drugs target only a single mechanism for the treatment of the complex pathophysiology of NDs. Hence, preparing a hybrid with carbazoles and FDA-approved drugs would expand their neuroprotective spectrum and would be a promising approach in drug discovery. The molecular mechanisms of action of most of the bioactive compounds are known within limitations. Henceforth, additional studies are necessary to fill this knowledge gap. Approaches based on the opportunities lying in untapped phyto-carbazole alkaloids (like murrayaine, girinimbine and mahanine) in the field of neuroprotection should also be explored. Other phyto-carbazole alkaloids that have revealed significant neuroprotective activity should be subjected to clinical trials to further assess their potential as a new drug or as pharmacophores for the development of a new generation of therapeutic agents.

To conclude, the phyto-carbazole alkaloids from the Rutaceae family were proven to be promising candidates in both the drug designing by natural sources and the development of nutraceuticals. We believe that the current review provides a colloquium of information that will help future investigation on the use of underutilized phyto-carbazole alkaloids to discover new neuroprotective drugs with the aid of animal models and clinical trials.

## Figures and Tables

**Table 1:** Neuroprotective mechanism displayed by Rutaceae family plant extracts and phyto-carbazole alkaloids.

Plant/Carbazole Alkaloid	Mechanism of Neuroprotection	Study	Refs
*Murraya koenigii* leaf (MKL)	Increased ACh Increased GPx, GSH, GRD, SOD, CAT Decreased LPO, NO Anti-amnesic	in vivo: mice, rat	[37,39,40]
Total alkaloidal extracts of MKL	AChE inhibition BACE1 inhibition Antioxidant activity	in vivo: mice	[36]
Mahanimbine from *Murraya koenigii*	AChE inhibition BACE1 inhibition Antioxidant activity Decreased ROS, MDA Nrf2-dependent induction of antioxidant enzymes Increased CAT, GRD, SOD, CAT Decreased NF-κB, TNF-α, IL-1β Decreased Aβ accumulation	in vitro: SK-N-SH cells, in vivo: mice	[39,44,45,46,47,48,49]
Girinimbinefrom *Murraya koenigii*	COX-2 inhibitor Decreased NF-κB, TNF-α, IL-6, IL-1β, iNOS Decreased MDA Increased GSH	in silico, in vitro: MDA-MB-231	[53,55]
Murrayanine from *Murraya koenigii*	Decreased iNOS and COX-2, PGE2, NO, I-κβ phosphorylation and NF-κB	in vitro: RAW 264.7 cells	[51,52]
Murrayanol from *Murraya koenigii*	Anti-inflammatory	in vitro:	[39]
Murrayamine-E from *Murraya koenigii*	Increased neurite outgrowth	in vivo: mice	[40]
Phyto-carbazole alkaloids from *Murraya tetramera* and *Murraya kwangsiensis*	Decreased NO	in vitro: BV-2 cells	[42,43]
*Clausena lansium* peel extract	Antioxidant activity Increased survival of neurons Decreased oxidative stress	in vivo: rat	[60]
Prenylated carbazole alkaloids	Decreased NO	in vitro: RAW 264.7 cells	[56,57]
Clauemarazole E clausine K and clausine from the stems of *Clausena emarginata*	Decreased NO	in vitro: BV-2 cells	[58]
Phyto-carbazoles from *Clausena dunniana*	Decreased NO	in vitro: BV-2 cells	[59]
Hydroethanolic extract of the stems and leaves of *Clausena lenis*	Neuroprotection against 6-OHDA induced apoptosis	in vitro: SH-SY5Y	[62]
Clausenalenine A and its analogues from *Clausena lenis* fruits	Neuroprotection against 6-OHDA induced apoptosis	in vitro: SH-SY5Y	[62]
Clausine Z from *Clausena excavata*	CDK5 inhibitor	in vitro	[63]
*O*-Demethylmurrayanine, clausine D, carbazole-3-carboxylate from the roots of *Clausena lansium*	Decreased superoxide anion	in vitro	[57]
Geranylated phyto-carbazole alkaloids (clauselansiumines A–B) along with other carbazole alkaloids from stem and leaves of *Clausena lansium*	Neuroprotection against 6-OHDA induced apoptosis	in vitro: SH-SY5Y	[70,71]
Methanolic extract of *Glycosmis pentaphylla*	AChE inhibition BChE inhibition Antioxidant activity	in vitro	[85]
Total alkaloid extract of *Glycosmis pentaphylla*	Antioxidant activity	in vitro	[86]
Zanthoaustrones A–C from the roots of *Zanthoxylum austrosinense*	Decreased NO	in vitro	[95]
*Zanthoxylum piperitum* *Zanthoxylum schinifolium*	Antioxidant activity Protect neuronal cells from high glucose–induced cytotoxicity	in vitro	[98,101]
Methanolic and Ethyl acetate extracts of Zanthoxylum capense root	Caspase-3 inhibition Decreased ROS	in vitro: SH-SY5Y	[102]
*Zanthoxylum *bungeanum** extracts (water, volatile oil)	Increased Nrf2, HO-1 Increased PI3K/Akt, Bax Reduced Bcl2 Increased CAT, SOD, GSH Decreased MDA Antioxidant activity	in vivo: mice	[105]
*Zanthoxylum fagara* *Zanthoxylum rhoifolium* *Zanthoxylum monophylla* *Zanthoxylum quinduensis* *Zanthoxylum davyi*	AChE inhibition	in vitro	[113,114]
Hydroethanolic extract of *Zanthoxylum alatum*	AChE inhibition TNF-α, IL-1 β and IL-10 inhibition Down-regulation of *NF*κ*B*, *Tau*, Bax, Caspase-3 Up-regulation of *Nrf2*, *HO-1*, *PP2A, BDNF*	in vivo: rat	[115]
